# Stable isotope signatures and nutritional sources of some dominant species from the PACManus hydrothermal area and the Desmos caldera

**DOI:** 10.1371/journal.pone.0208887

**Published:** 2018-12-17

**Authors:** Xiaocheng Wang, Chaolun Li, Minxiao Wang, Ping Zheng

**Affiliations:** 1 Key Laboratory of Marine Ecology and Environmental Sciences, Institute of Oceanology, Chinese Academy of Sciences, Qingdao, P. R. China; 2 Laboratory for Marine Ecology and Environmental Science, Qingdao National Laboratory for Marine Science and Technology, Qingdao, China; 3 National Marine Environmental Monitoring Center, Dalian, China; 4 University of Chinese Academy of Sciences, Beijing, P. R. China; 5 Center for Ocean Mega-Science, Chinese Academy of Sciences, Qingdao, P. R. China; Fred Hutchinson Cancer Research Center, UNITED STATES

## Abstract

Deep-sea hydrothermal vents in the western Pacific are increasingly explored for potential mineral extraction. The study of the composition of the food web plays an important guiding role in the ecological protection and restoration of potential mining areas. The general picture of the nutritional sources of species should be established to assess the potential impacts of future mining activities on the biological composition and food sources. To provide basic information, we analyzed the carbon and nitrogen stable isotope ratios of the dominant macrofauna (mussels, commensal scale worms, crustaceans, gastropods, and vestimentiferans) at three different sites in the PACManus hydrothermal area and the Desmos caldera. The δ^13^C ratio was significantly different between species: mussels and commensal scale worms showed lighter δ^13^C ratios, whereas crustaceans showed heavier ratios. In terms of δ^15^N, mussels had the lowest values and the crustaceans had the highest values. By taking into account these stable isotope signatures, we were able to develop inferences of the food sources for vent community organisms. We found that the food web was based on various species of chemoautotrophic bacteria. Mussels appeared to rely primarily on sulfur-based endosymbionts, which use the Calvin–Benson–Bassham (CBB) cycle and RuBisCO form I as the CO_2_-fixing enzyme. Commensal polychaetes mostly obtained their nutrition from their hosts. Crustacean species were omnivorous, feeding on chemosynthetic bacteria, sedimentary debris, or even animals according to the local environment. In contrast, gastropods relied mainly on symbiotic bacteria with some supplementary consumption of detritus. Vestimentiferans obtained food from symbiotic bacteria using the RuBisCO form II enzyme in the CBB cycle and may have several symbionts using different fixation pathways. Although most macrofauna relied on symbiotic chemoautotrophic bacteria, our study suggested a closer trophic relationship between animals. Therefore, to evaluate the potential impacts of deep sea mining, it is necessary to study the cascade effects on the food web of the whole ecosystem. Before exploiting deep-sea resources, further systematic investigations concerning the protection of deep-sea ecosystems are necessary.

## Introduction

Hydrothermal vents occur in geologically active seafloor areas, such as mid-ocean ridges and volcanic hotspots [1, 2]. Geothermally heated fluids emitted from the seafloor are rich in reduced gases and metals [3], and dense invertebrate communities thrive in the areas where these fluids mix with colder and oxygenated seawater [4]. Most hydrothermal deposits containing significant amounts of metals are of economic interest to mining companies [5]. Almost 20% of all known global vent fields currently fall within mining exploration leases [6], so basic information on the diversity and connectivity of vent systems is necessary for establishing effective environmental management framework[7].

The stability of the food web is one of the most important factors affecting ecological stability. There are complicated food relationships between different species to maintain stability. Understanding the food sources of the dominant species is a crucial step determining the food web and nutritional relationships. Oceanwide, several inorganic carbon sources underpin various ecosystems. Because deep-sea vent communities exist so far outside the photic zone and the ordinary photosynthetic basis for many food webs is unavailable, questions regarding the nutritional resources of those species have sustained scientific interest since their discovery [8]. The main carbon and nitrogen sources for hydrothermal vent fauna have been identified as being primarily of local origin rather than being imported from photic zone communities [9, 10]. As with surface systems, carbon dioxide and methane are the primary carbon sources utilized by autotrophic organisms [11, 12], but these are fixed by chemoautotrophic bacteria rather than organisms utilizing photic energy. Such bacteria form the base of the food chain, oxidizing reduced materials in vent fluids and utilizing the energy released to fix carbon [13–15]. Vent community invertebrates always inhabit with these bacteria that exist freely or symbiotically as endobionts which can be extracellular or intracellular, or as epibionts [16]. Animals derive the vast majority of their nutrition from these relationships [17–19]. Epipelagic photosynthetic primary production may also support part of the trophic system, depending on the environmental characteristics of the vent system. Some upper sublittoral vents and upper bathyal vents have a mixed photosynthetic-chemosynthetic system, with photosynthetically–derived organic matter as a nutritional source [20–23].

Stable isotope analysis is a useful approach for investigating such trophic interactions, evaluating community structure, and examining the trophodynamics of ecological communities [18, 24, 25]. The δ^13^C isotopic signature is a good indicator of consumed food sources since carbon trophic discrimination is small, ranging from 0 to 1.5 ‰ between successive trophic levels [26]. The ratio of the stable isotopes of nitrogen (δ^15^N) can be used to estimate trophic position, with typical enrichment in δ^15^N increasing by 3 to 4 ‰ per trophic level [27, 28].

Carbon origins are distinct stable isotope ratios that are useful for determining the origin of energy. In surface layers, stable isotopes of dissolved carbon dioxide are relatively constant at approximately 0 ‰ [28]. The withdrawal of carbon to form carbonates involves little isotopic fractionation, whereas the uptake of dissolved inorganic carbon during planktonic photosynthesis involves a larger kinetic fractionation that results in algal values of approximately –19 ‰ to –24 ‰ [28]. Particulate organic matter (POM) in the oceans predominantly reflects a marine planktonic origin. However, POM could also reflect microbial signal (bacteria-archaea) at vent systems, especially in the vent plume or close to the vent emissions. Biological fractionation, the input of organically produced material, its oxidation, and changes in temperature and pH all influence the composition of isotopes [29, 30]. δ^13^C values in dissolved inorganic carbon (DIC) are generally lower at depth compared to the surface water layers [29, 31], in some places by -9 ‰ [32]. Methane is generally the main inorganic carbon source at cold seep fields [33], with values ranging from -60 ‰ to -110 ‰ [32, 34–36]. The type of inorganic carbon source used by producers in hydrothermal vent ecosystems can be estimated according to stable isotope signatures. The carbon isotopic ratios of animal tissues are lower or equal to -40 ‰when they used methane-based energy sources [13]. These signatures have been exploited in a number of previous trophic studies of hydrothermal vent communities around the world [18, 22, 24, 25, 37, 38]. Most recently, stable isotope ratios have been used to examine the relationships within the trophic network in the Guaymas Basin [39], revealing variable nutritional pathways during the life time of the hydrothermal vent snail *Ifremeria nautilei* and barnacle *Eochionelasmus ohtai manusensis* from the Manus Basin, Western Pacific [40].

**Fig 1:**
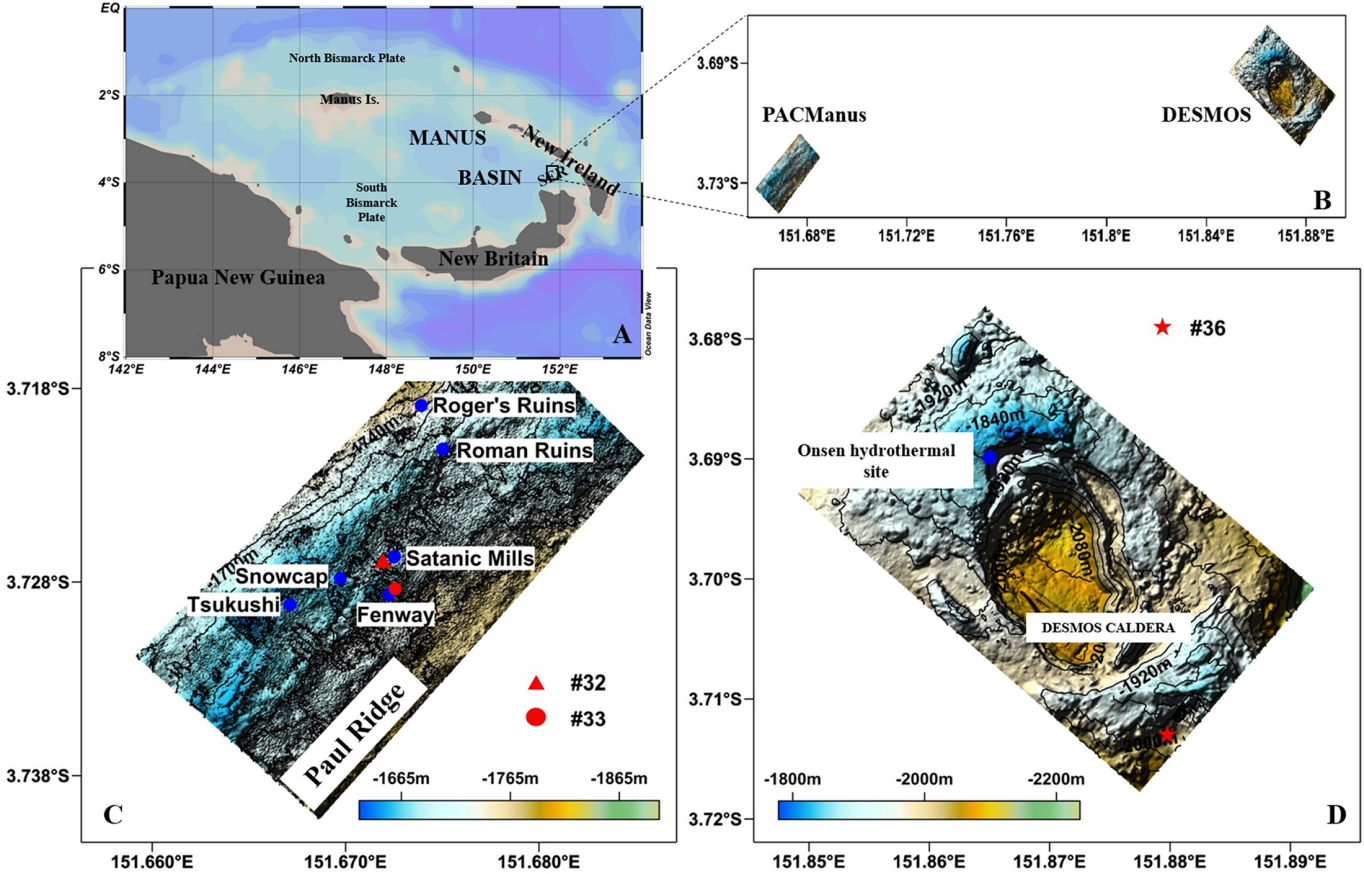
Location and microbathymetry of the PACManus hydrothermal vent area and the Desmos caldera. (A) Regional location map of the research area. (B) The relative location of research areas, partly republished from [46] under a CC BY license, with permission from [Elsevier], original copyright [2017]. (C) Microbathymetry of the PACManus developed from multibeamsonar data of the ROV *Faxian*, republished from [46] under a CC BY license, with permission from [Elsevier], original copyright [2017]. (D) Microbathymetry of the Desmos caldera developed from multibeamsonar data of the ROV *Faxian*.

The Eastern Manus Basin (EMB), from the Bismarck Sea, north-east of Papua New Guinea, Western Pacific, is the location of a number of hydrothermal areas, including the Southeast Ridges region (SER) (Fig 1). The SER is a rift zone of pre-existing island arc crust that contains several sigmoidally–shaped volcanic ridges [41]. Both the Papua-Australia-Canada-Manus (PACManus) and the Desmos caldera hydrothermal areas are located in this region. The PACManus site is a polymetallic type of mineral deposit consisting of sphalerite, chalcopyrite, bornite, wurtzite, pyrite, marcasite, enargite, tennantite, galena, Pb-As-sulfosalt, gold, covellite, digenite and chalcocite [5]. The Onsen site at the Desmos caldera has a typical acid-sulfate type of mineralization consisting of enargite, covellite, chalcopyrite, pyrite and marcasite [5]. Both vent fields are considered important sources of potential mineral wealth, and there is basically no information on the trophic relationships in the community. In these areas, a steady discharge of vent fluid supports well-developed biological communities, including bacterial mats, molluscs, tube worms, crabs, anemones, holothurians, crustaceans and fishes [42–45]. The species composition is similar across the Manus Basin fields, but the relative abundance varies from field to field, possibly as a result of the amount of time the communities have existed [43]. Overall, the vent fauna displays closer affinity with the communities of the North Fiji and Lau Basins than the Mariana Trough vent region [43].

This work presents the trophic structures of some dominant assemblages from the PACManus hydrothermal area and the Desmos caldera using stable isotope analysis. Specifically, our aims were: to provide descriptive information about the stable isotope signatures of some dominant species and to investigate potential food sources and nutritional relationships within the communities in order to lay the groundwork for subsequent studies and mineral extraction.

## Materials and methods

### Sample collection and processing

**Fig 2:**
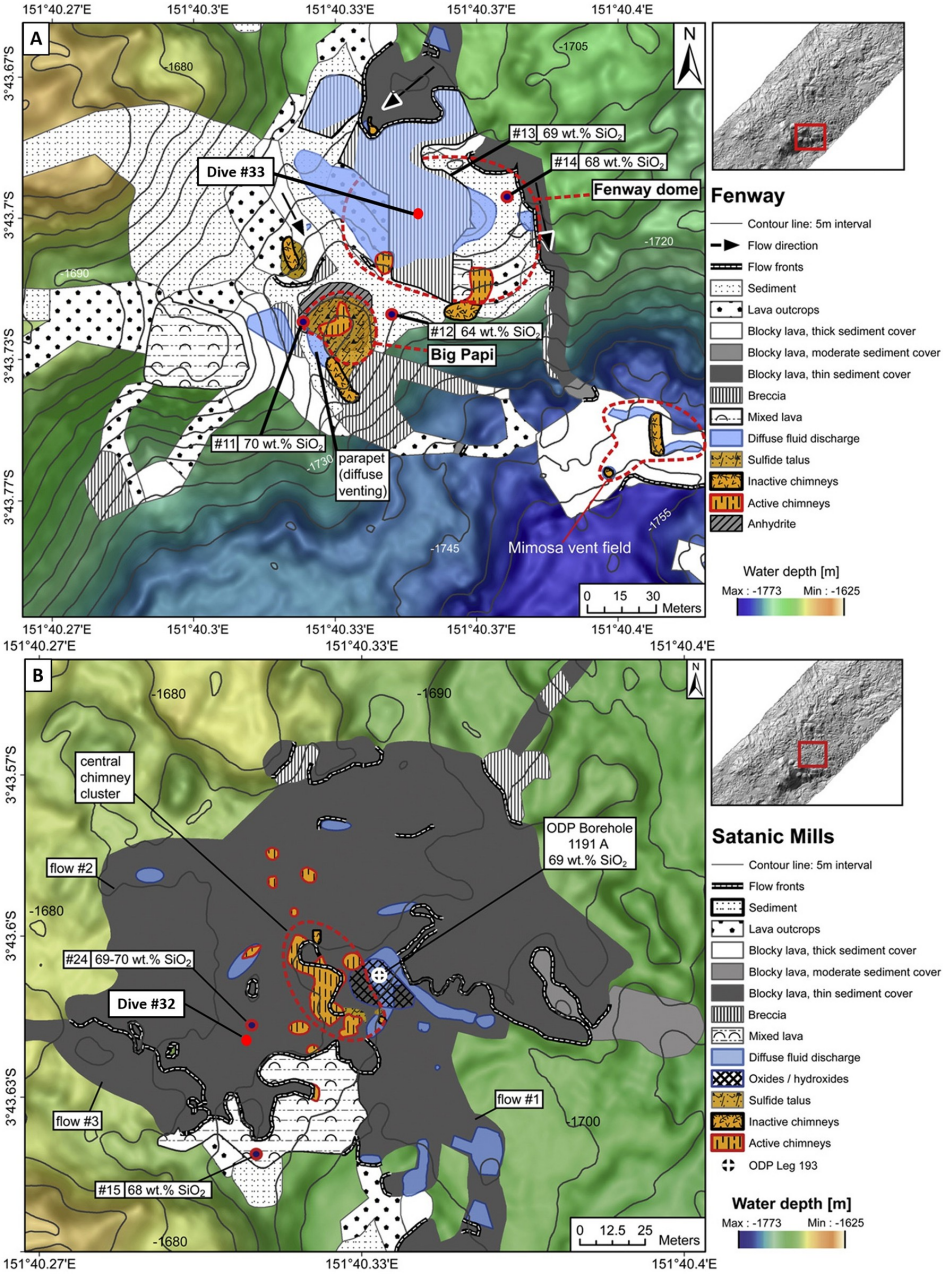
Geologic maps of the Fenway and Satanic Mills hydrothermal fields. (A) Geologic map of the Fenway hydrothermal field, showing all mapped seafloor structures republished from [41] under a CC BY license, with permission from [Elsevier], original copyright [2014]. The red dot represents the sampling site. (B) Geologic map of the Satanic Mills hydrothermal field showing all mapped seafloor structures republished from [41] under a CC BY license, with permission from [Elsevier], original copyright [2014]. The red dot represents the sampling site.

A research cruise to the PACManus hydrothermal area and the Desmos caldera was attended in June 2015 by the R.V. *Kexue*, and sampling at depth was conducted using the remotely operated vehicle (ROV) *Faxian* belonging to the Chinese Academy of Sciences. Samples were collected from three sites several meters away from vents, and general information about these sites is shown in Fig 1 and Table 1. Permission from the ministry of foreign affairs of Papua New Guinea was obtained. Site Dive #32 was located approximately 25 m from the edge of the central chimney cluster in the Satanic Mills field of PACManus (Figs 1C and 2B) at 1693 m depth. A cluster of active black chimneys was observed in the sampling field. Numerous large gastropods were present on the blocky lava along with associated crustaceans and crabs. Site Dive #33 was in the center of the Fenway dome (Figs 1C and 2A) at 1724 m depth. Scattered black active chimneys were observed around the sampling site. Numerous fauna, including molluscs, clusters of vestimentiferans, anemones, and crustaceans were present on the breccia. Site Dive #36 (Fig 1D) was situated southeast of Desmos caldera at 1912 m depth. Several low-temperature seeps (~1 °C) and inactive chimneys were observed around the sampling field. Abundant crustaceans were present on and between small pieces of rock.

**Table 1:** General description of three sampling sites from the PACManus hydrothermal area and the Desmos caldera.

Site	Depth (m)	Latitude	Longitude	Salinity	Description	Communities
Dive #36	1912	3°42.24′ S	151°52.63′ E	35.5	Several low-temperature seeps (~1 °C) and inactive chimneys	Abundant crustaceans (*Munidopsis lauensis*, *Rimicaris vandoverae*, etc.)
Dive #32	1693	3°43.62′ S	151°40.32′ E	35.7	A cluster of active black chimneys	Numerous large gastropods (*Ifremeria nautilei*, etc.), crustaceans (*Munidopsis lauensis*, *Rimicaris vandoverae*, etc.), and crabs
Dive #33	1742	3°43.70′ S	151°40.35′ E	35.7	Scattered black active chimneys	Numerous fauna, including molluscs (*Bathymodiolus manusensis*, etc.), clusters of vestimentiferans (*Arcovestia ivanovi*, etc.), anemones, and crustaceans

We collected a large number of specimens at each site. The squat lobster *Munidopsis lauensis* and the shrimp *Rimicaris vandoverae* were collected using a suction pump coupled to a rotating carousel of acrylic collection bottles. The gastropods *Ifremeria nautilei* (Site Dive #32) and *Provanna nassariaeformis* (Site Dive #36), the mussel *Bathymodiolus manusensis*, and the vestimentiferans species *Arcovestia ivanovi* were collected using a scoop controlled by an ROV arm and brought to the surface in a closed and thermally insulated biobox. The commensal scale worms *Branchipolynoe* spp. were found in the mantle cavity of *B*. *manusensis*. Samples were preserved on ice immediately upon arrival on board. Three or five voucher specimens of each species were preserved in a seawater-formalin solution for further taxonomic study. Three or more samples of each species were processed for stable isotope analysis. The gills and feet of *B*. *manusensis*; the tentacles, foreparts, and trunk of *A*. *ivanovi*; and the muscles of the gastropods and crustaceans *M*. *lauensis* and *R*. *vandoverae* were dissected. Muscle tissue was selected for nutritional analysis, and other tissues were used for comparative analysis. After separation, the tissue samples were rinsed with deionized water to remove any residual seawater and frozen at -80 °C.

POM samples for isotope analysis were collected from three water depths (a surface layer at 0 m, a mid-water layer at 800 m, and a demersal layer at 1637 m) using Niskin water bottles mounted on the ROV above the sampling site at Site Dive #32. Approximately 2 L of water was passed through a 200 μm sieve to exclude large prey items and then filtered through GF/F filters of 0.70 μm pore size that had been pretreated at 450 °C for 4 h. The filters were frozen at -80 °C after filtration.

In the laboratory, all faunal samples for stable isotope analysis were freeze-dried and homogenized in an agate mortar. One milligram of tissue was placed in tin capsules for carbon and nitrogen isotope analyses. Particulate organic matter samples were separated from filters and a subset was acidified to remove inorganic carbon and measure the δ^13^C signature of organic carbon only. Acidification was carried out by adding drops of 0.1 M HCl until effervescence ceased. The sample was then dried at 60 °C under a fume extractor to evaporate the acid. To prevent the loss of dissolved organic matter, samples were not rinsed [39].

### Stable isotope analysis

The isotopic compositions were analyzed using an elemental analyzer (Flash EA 1112Ht, Thermo Fisher Scientific, Inc., San Diego, CA, USA) coupled with an isotope-ratio mass spectrometer (Finnigan Delta V Advantage, Thermo Fisher Scientific, Inc.). Stable isotope ratios are expressed in δ (‰) notation with respect to Pee Dee Belemnite (PDB) for δ^13^C and atmospheric N_2_ for δ^15^N

$$\delta X(‰)=[(R_{sample}/R_{standard})-1]\times 10^{3}$$

where X is either ^13^C or ^15^N, R_sample_ is the ^13^C/^12^C or ^15^N/^14^N isotope ratio in the sample and R_standard_ is the ^13^C/^12^C or ^15^N/^14^N isotope ratio for the standard. An internal standard (glycine) was run for every twelve samples. Measurement precision was 0.1 ‰ and 0.2 ‰ for δ^13^C and δ^15^N values, respectively.

### Statistical analysis

The normal distribution of data was confirmed using the one-sample Kolmogorov-Smirnov Test. The homogeneity of variance was assessed using Levene's test. One-way ANOVA was performed to assess the significance of differences in δ^13^C and δ^15^N values across different species and different tissues when the data fits normal distribution and homogenous variance. A Kruskal-Wallis H-test was used when the data did not approach normality or homogeneity of variance. Multiple parametric comparisons were performed using the S-N-K multiple range test. A Mann-Whitney U-test was used when the data lacks normal distribution or homogeneous variance. SPSS Version 19 was used for all statistical analyses.

## Results

### Stable isotope ratios in individual tissues and POM

**Fig 3:**
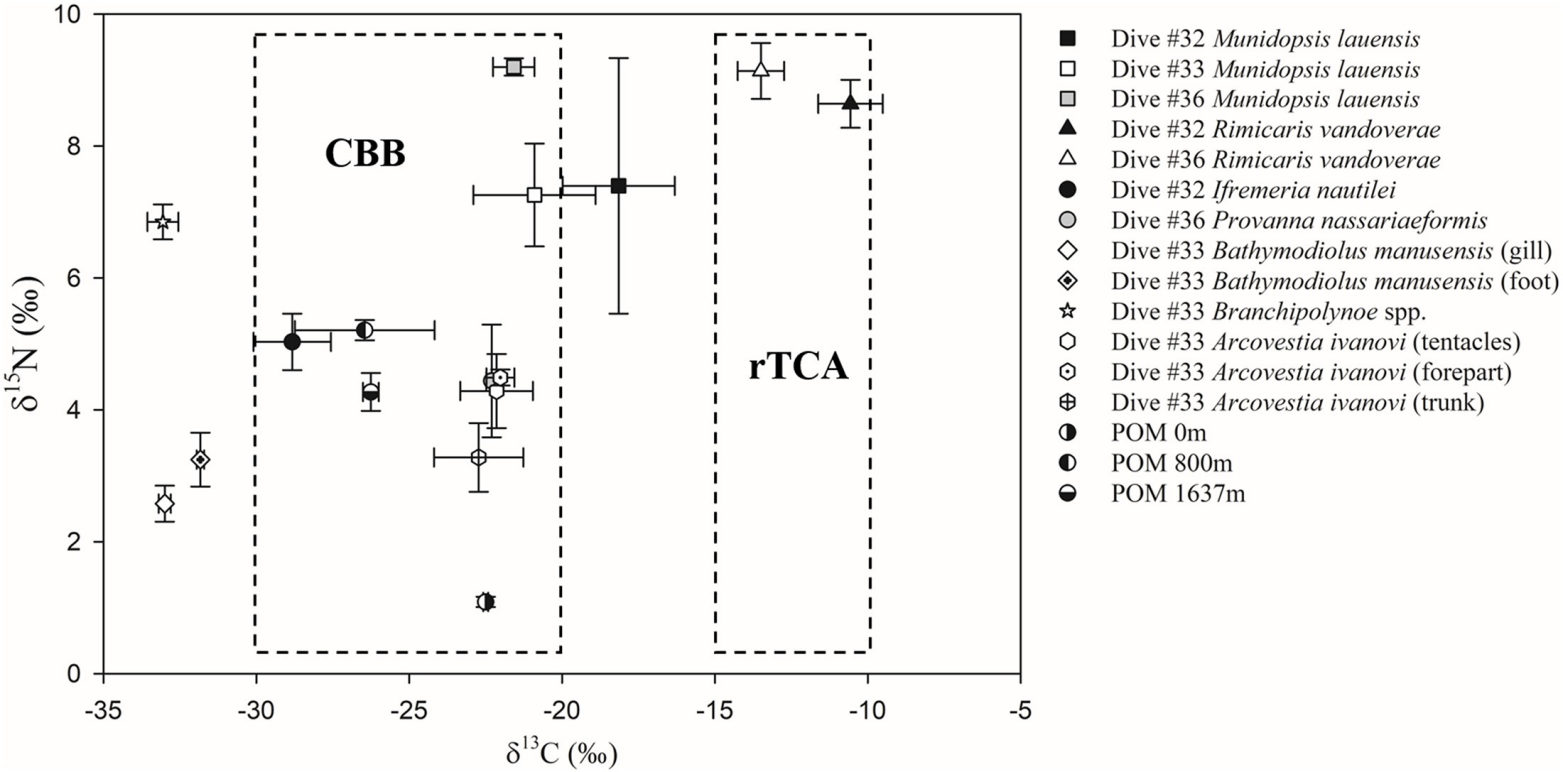
Average δ^15^N vs. δ^13^C values for the dominant species and POM from the PACManus hydrothermal area and the Desmos caldera. Dotted boxes represent different types of carbon fixation.

The full suite of results is presented in Table 2. The δ^15^N of all taxa and POM were plotted against the corresponding δ^13^C (Fig 3). The δ^13^C value of POM in the surface water layer was the highest (-22.50 ‰), followed by the values of POM collected at 1637 m (-26.26 ‰) and 800 m (-26.46 ‰). The δ^15^N value of POM in the surface water layer was the lowest (1.09 ‰), followed by the values of POM collected at 1637 m (4.27 ‰) and 800 m (5.21 ‰).

**Table 2:** The δ^13^C and δ^15^N values (‰) for the species collected from the PACManus hydrothermal area and the Desmos caldera.

Site	Species	Taxa	δ^13^C			δ^15^N			n
			Min	Max	Mean ± SD	Min	Max	Mean ± SD	
Dive #36	*Munidopsis lauensis*	crustacean	-22.96	-20.70	-21.58 ± 0.678	9.01	9.40	9.20 ± 0.131	10
	*Rimicaris vandoverae*	crustacean	-14.93	-12.32	-13.50 ± 0.759	8.33	9.85	9.14 ± 0.423	14
	*Provanna nassariaeformis*	gastropod	-22.43	-22.18	-22.30 ± 0.175	3.84	5.04	4.44 ± 0.854	2
Dive #32	*Munidopsis lauensis*	crustacean	-21.76	-16.16	-18.15 ± 1.835	4.55	10.02	7.40 ± 1.937	7
	*Rimicaris vandoverae*	crustacean	-12.85	-9.11	-10.57 ± 1.056	8.04	9.32	8.64 ± 0.363	10
	*Ifremeria nautilei*	gastropod	-29.94	-27.45	-28.83 ± 1.267	4.71	5.52	5.03 ± 0.428	3
	POM 0 m		-22.58	-22.41	-22.50 ± 0.087	1.00	1.15	1.09 ± 0.078	3
	POM 800 m		-26.54	-26.38	-26.46 ± 0.081	5.08	5.38	5.21 ± 0.153	3
	POM 1637 m		-26.52	-26.01	-26.26 ± 0.257	4.05	4.59	4.27 ± 0.286	3
Dive #33	*Bathymodiolus manusensis* (gill)	mussel	-33.12	-32.41	-33.00 ± 0.198	2.32	3.22	2.58 ± 0.273	11
	*Bathymodiolus manusensis* (foot)	mussel	-31.96	-31.57	-31.83 ± 0.121	2.19	3.79	3.25 ± 0.409	11
	*Munidopsis lauensis*	crustacean	-23.73	-18.10	-20.90 ± 2.000	5.88	8.14	7.26 ± 0.780	10
	*Arcovestia ivanovi*(tentacles)	vestimentiferan	-22.99	-21.30	-22.14 ± 1.189	3.89	4.68	4.29 ± 0.561	2
	*Arcovestia ivanovi*(forepart)	vestimentiferan	-22.34	-21.70	-22.02 ± 0.457	4.40	4.58	4.49 ± 0.121	2
	*Arcovestia ivanovi* (trunk)	vestimentiferan	-23.76	-21.70	-22.73 ± 1.461	2.91	3.65	3.28 ± 0.521	2
	*Branchipolynoe* spp.	polychaete	-33.03	-32.58	-33.06 ± 0.507	6.49	7.11	6.85 ± 0.265	4

The gastropod *I*. *nautilei* and two crustaceans were collected at Site Dive #32. The gastropod was found in the middle of the animal clusters and on the stacks of shrimps. The ratios of *I*. *nautilei* were approximately -28.83 ‰ for δ^13^C and 5.03 ‰ for δ^15^N. The ratios of the two crustaceans *M*. *lauensis* and *R*. *vandoverae*, were higher than those found on gastropods. *R*. *vandoverae* had the highest ratios, which were approximately -10.57 ‰ for δ^13^C and 8.64 ‰ for δ^15^N.

For the taxa at Site Dive #33, the mussels *B*. *manusensis* and commensal worms *Branchipolynoe* spp. had lower δ^13^C values, and the mussels had the lowest δ^15^N values. The crustaceans *M*. *lauensis* had the highest δ^13^C and δ^15^N values, while other animals showed intermediate signals. The values of δ^13^C and δ^15^N differed significantly between the gill and foot tissues of the mussels (*p* < 0.05). The mussels displayed little range, from -33.12 ‰ to -32.41 ‰ for δ^13^C and from 2.32 ‰ to 3.22 ‰ for δ^15^N in gills and from -31.96 ‰ to -31.57 ‰ for δ^13^C and from 2.19 ‰ to 3.79 ‰ for δ^15^N in foot tissue. The commensal scale worms were found in the mantle cavity of the mussels. The δ^13^C values did not differ between the worms and the mussel gill tissues, but a significant difference was present between the worms and the foot tissues of the hosts (*p* < 0.05). The values of δ^15^N in scale worm tissues were, on average, 3.6 ‰ higher than the values in the foot tissues of the host. For the vestimentiferans *A*. *ivanovi*, the δ^13^C values of all three parts were approximately –22 ‰ and notably high compared to the values of the mussels and commensal scale worms. In δ^15^N, however, the values in the trunk were especially low among the three parts, which were similar to the values of the mussels’ foot. The values in the other two parts, tentacles and foreparts, were in the range of δ^15^N in mussels and the commensal scale worms. Although *A*. *ivanovi* vestimentiferans were collected in association with the mussels, no statistical comparison was possible because of the limited number of specimens.

The gastropod *P*. *nassariaeformis* and two crustaceans were collected at Site Dive #36. The ratios of *P*. *nassariaeformis* were approximately -22.30 ‰ for δ^13^C and 4.44 ‰ for δ^15^N. *R*. *vandoverae* showed the highest ratios of δ^13^C. The ratios of δ^15^N were similar between these two crustaceans.

*M*. *lauensis* crustaceans were collected from all three sampling sites and *R*. *vandoverae* crustaceans were collected at Site Dives #32 and #36. Both species showed higher values of δ^13^C and δ^15^N, with significant differences in these measures between fields (*p* < 0.05). The δ^13^C and δ^15^N values of *M*. *lauensis* ranged from -23.73 ‰ to -16.16 ‰ and from 4.55 ‰ to 10.02 ‰, respectively. The values of δ^15^N were highest at Site Dive #36, followed by Site Dive #32 and Site Dive #33. The δ^13^C and δ^15^N values for *R*. *vandoverae* ranged from -14.93 ‰ to -9.11 ‰ and from 8.04 ‰ to 9.85 ‰, respectively. The values of δ^13^C were higher at Site Dive #32, followed by Site Dive #36. The values of δ^15^N were higher at Dive #36, followed by Site Dive #32.

## Discussion

### Potential carbon sources and POM

Generally, methane and carbon dioxide are the two common carbon sources at hydrothermal vents. The carbon sources of vent species could be inferred according to the carbon isotopic ratios in tissues. Carbon isotopic ratios of animal tissues were lower or equal to -40 ‰ when they used methane-based energy sources. When animals used heterotrophic and sulfur-based energy sources, the carbon ratios of tissues were above -40 ‰ [33]. In addition, the dual symbiosis of thiotrophic and methanotrophic bacteria occurred in many *Bathymodiolus* species, whose isotopic values were related to the proportion of different bacteria present [47, 48]. Moreover, methane oxidation occurs less frequently in the basic carbon fixation process, although it is energetically comparable to sulfide in oxidation potential [35, 49]. Because metazoans are unable to bind and store methane, species with endosymbiotic methanotrophic bacteria may thrive only in environments with stable and elevated methane levels [35]. Our data, which were all above -40 ‰ for δ^13^C in tissues, suggest that the main carbon source for all sampled animals in our study was carbon dioxide, which is consistent with previous findings showing that sulfide oxidation was the primary energy acquisition pathway at hydrothermal vents [50, 51].

Essentially, two distinct carbon isotopic signals existed at the vents: one group with a composition of approximately -10 ‰ to -15 ‰, which usually included shrimps and vestimentiferans, and a second group with an isotopic signal of -20 ‰ to -30 ‰, which usually included mussels [25, 52, 53]. This strongly suggests that the use of carbon fixation pathways by chemoautotrophic bacteria differs between the two groups [52, 54]. Heavier values can be explained by the operation of the reverse tricarboxylic acid (rTCA) cycle, with a fractionation of 2 ‰ to 14 ‰ [55–57]. The lighter values can be explained by the operation of the Calvin-Benson-Bassham (CBB) cycle. This pathway includes two types according to different forms of RuBisCO. Here, the fractionation by bacteria using RuBisCO form I ranges from 22 ‰ to 33 ‰ [53, 58, 59], whereas the fractionation by RuBisCO form II ranges from 18 ‰ to 22 ‰ [53]. Compared with the two carbon fixation pathways, fractionation via the rTCA cycle contributes very little, resulting in a higher δ^13^C value in chemoautotrophic bacteria. In contrast, the δ^13^C value is lower in bacteria using the CBB cycle.

Among the isotopic values of the POM in the three water layers in our study, the δ^13^C values of the POM at the surface layer were high, while the δ^15^N values were low, reflecting the abundance of phytoplankton. Photosynthetic primary production in phytoplankton takes place using carbon dioxide as a carbon source in the photic zone, placing phytoplankton at the bottom of the food web with the lowest δ^15^N values compared to other organisms [26, 27]. The δ^15^N values of POM at 800 m were the highest, likely due to the presence of detritus or remains because of the higher δ^15^N of animals at higher trophic levels [27]. The δ^15^N values of the deep sea at 1637 m decreased compared to the middle layer, possibly due to a mixture of materials from the hydrothermal vent fields and detritic fall from the upper water layers. Because POM was mainly made up of chemoautotrophic bacteria which had lowest δ^15^N values as primary producers at vent ecosystems, the values may be situated between the values of chemoautotrophic bacteria and detritic fall.

### Mussels and commensal scale worms

Mussels are common in hydrothermal fields and always thrive on the bacterial mats or rocks in overflow areas [42, 60, 61]. Although mussels’ nutrition is primarily obtained through the productivity of endosymbionts [9, 13], POM may also be a component of their diets due to their filtration ability [62, 63]. In our samples, the isotopic signatures of foot tissues can be regarded as reflective of individual nutritional conditions over a long time period, while the values obtained from gill tissues more likely represent a mixture of individuals’ and endosymbionts’ values due to the high number of bacteria hosted there [13]. Our findings demonstrated that the average carbon value was -31.83 ‰ in foot tissues, which was in the range of organic carbon derived from seawater CO_2_ fixed via sulfide oxidation (-42 ‰ to -30 ‰) in mussels and differed significantly from mussels harboring exclusively methanotrophic bacteria endosymbionts [32, 33]. It is possible that the nutritional sources for *B*. *manusensis* are primarily from sulfur-based endosymbionts. Because mussels rely on endosymbionts, and the carbon isotopic fractionation between the contiguous levels was small [26], we can infer that the carbon signature of endosymbionts approximates the signature in gills containing numerous bacteria, whose average carbon value was -33 ‰.

The δ^13^C values of mussels in our study were in the range of the light carbon isotopic group, suggesting that the pathway of the symbiotic bacteria in mussels is primarily via the CBB cycle with RuBisCO form I as the CO_2_-fixing enzyme [25]. This further indicated that the δ^13^C value of dissolved inorganic carbon (DIC) could be calculated according to the average carbon value of gills, which was -33 ‰, and the fractionation by bacteria using RuBisCO form I which ranged from 22 ‰ to 33 ‰ in the local environment. The δ^13^C values of DIC may range from -11 ‰ to 0 ‰, varying between individual mussels. Additionally, some undigested food debris was observed in the gut contents during the dissection, which could indicate filter-feeding. However, the carbon isotopic values of the foot tissue were closer to those of gills rather than the POM values. It could be suggested that even if there was an input of filter-feeding, it was low.

We observed that the δ^13^C isotopic composition of the commensal worm *Branchipolynoe* spp. was very similar to that of its host mussels, but its δ^15^N values were higher, from 3.6 ‰ to 4.3 ‰ than those of foot tissues in the mussels. These were in the range of accepted δ^15^N enrichment per trophic level (3 to 4 ‰) in agreement with previous studies [25, 39, 64, 65]. This suggests that the worms’ food is sourced mainly from its host.

### Crustaceans

Crustaceans were collected in considerable numbers at three sites in the cracks and rock surfaces. Vent crustaceans have been recorded as bacterial grazers, episymbiont hosts, scavengers or detritus feeders, or even predators [22, 24, 66–68]. In addition, some authors have suggested that crustaceans subsist largely on a bacterial diet; unlike mussels, crustaceans primarily feed on episymbiotic bacteria and bacterial mats [16, 39, 66–68]. For alvinocaridid shrimps, nutrition could be transferred from bacteria to the host through soluble bacterial products that occur by permeation across the gill chamber integument directly, or to a significant degree, via the digestive tract [69, 70]. However, much higher fractions of photosynthetic carbon may occur in some vent fields [69]. For crabs in the genus *Munidopsis*, most were not reliant upon nutrition from bacterial endosymbionts, but on a mixed diet based on sediment bacteria, metazoans associated with bacterial mats, or other animals including polychaetes, limpets, protozoans and crab larvae [25, 71, 72].

Our study found δ^13^C values ranging from -23.73 ‰ to -9.11 ‰, with *R*. *vandoverae* showing values from -14.93 ‰ to -9.11 ‰ and *M*. *lauensis* showing lower values from -23.73 ‰ to -16.16 ‰, which were both in the range of values found in previous studies about related species [18, 25, 73–75]. Compared to mussels, the values of crustaceans showed a much larger range, resulting from the omnivorous characteristics. According to the highest δ^15^N values for the crustaceans, compared to other animals at the same site, bacteria might be one of the food sources, but not the only one. Some sources with higher trophic levels than bacteria, such as POM and animals, should also be food sources. Among the three sites we studied, a mass of crustaceans was found at Site Dive #32 and Site Dive #36, whereas few mussels or vestimentiferans were found. As such, crustaceans from these two sites were not thought to feed on mussels or vestimentiferans. In addition, the δ^13^C values of *R*. *vandoverae*, which were only collected at Site Dive #32 and Site Dive #36, were isolated from other animals or potential food sources and were in the range of the heavy-value group, suggesting that bacteria with an rTCA pathway are the main nutritional source for the shrimps. In addition, the δ^13^C values of *M*. *lauensis* varied widely between individuals and was in the middle of the two groups, which may indicate that the carbon was fixed with Rubisco form II enzyme in the CBB cycle or in a mixture of the two cycles by the bacteria they consumed at these two sites.

At Site Dive #32, the two stable isotope values of the two crustaceans were significantly different, with the signatures of *M*. *lauensis* low and highly individual, whereas *R*. *vandoverae* showed relatively weak differences between individuals. The δ^15^N values of *M*. *lauensis* ranged from 4.55 ‰ to 10.02 ‰, which might suggest that they were more omnivorous. At Site Dive #36, the δ^13^C values of *M*. *lauensis* were still low, but the δ^15^N values were similar to those of *R*. *vandoverae*. They were at the same trophic level, but the food sources were different. The δ^13^C values of *M*. *lauensis* were closer to those of the gastropods, which might suggest that a proportion of *P*. *nassariaeformis* is the food source or that there is similar source between them. At Site Dive #33, the isotopic values of *M*. *lauensis* were similar to the same species from another two sites, which indicated similar food sources. In general, *M*. *lauensis* were more omnivorous. The high δ^15^N values indicated that they fed not only chemoautotrophic bacteria but also POM and animals.

### Gastropods

We collected two gastropod species in limited quantities. Compared with the crustaceans collected from Site Dive #32, the carbon and nitrogen isotope values of *I*. *nautilei* were low, which indicated a lower trophic level. On the basis of previous research showing symbiotic relationships of bacteria in gastropods, *I*. *nautilei* derived its nutrition from intracellular gill symbionts that can oxidize both sulfide and thiosulfate to fuel autotrophy [40, 76–78]. Methanotrophic bacteria may also be present, but in low abundance. The lower δ^15^N values in our results support that the source is from chemoautotrophic bacterial endosymbionts. The average δ^13^C value was -28.83 ‰, which suggested that sulfide-oxidizing bacteria were the dominant contributor. We suggested that the bacteria were likely utilizing the CBB cycle for carbon sequestration.

The carbon isotopic values of *P*. *nassariaeformis* from Site Dive #36 were similar to those of *M*. *lauensis*, whereas the nitrogen isotopic values were significantly lower at approximately 5 ‰. The genus *Provanna* is a characteristic gastropod of chemosynthetic faunas. Some of them may harbor symbiotic bacteria, but these species mainly feed on filamentous bacteria or detrital organic material [24, 79, 80]. From our results, the lower δ^15^N values indicated that they were at a low trophic level. Unlike another gastropod *I*. *nautilei*, the δ^13^C values of *P*. *nassariaeformis* were higher at approximately 6 ‰, which may indicate that their food sources were different.

### Vestimentiferans

Vestimentiferans were observed only at Site Dive #33, and there were only two individuals available for dissection. These are local species with food sources from endosymbiotic bacteria [23, 81]. The chemoautotrophic bacterial endosymbionts were mainly sulfur–oxidizing symbionts [16, 52]. Compared three parts of the vestimentiferans, there were no differences in δ^13^C values but significant differences in δ^15^N values. The signals in the trunk tissues were lowest. The δ^15^N values were lower than those of the crustaceans and higher than those of the mussels, which indicates a low trophic level. At Site Dive #33, the carbon fixation pathway of the symbiotic bacteria in vestimentiferans should be not the same as the mussels, resulting from a 10 ‰ higher δ^13^C value. The δ^13^C values of *A*. *ivanovi* were around -22 ‰, which were not in the heavier–value group and different from several previous results. This may resulted from the different types of symbiotic bacteria. Assuming that the vestimentiferans wholly rely on symbiotic bacteria, the different isotope signatures suggest that the symbionts primarily used RuBisCO form II enzyme in the CBB cycle to fix inorganic carbon, or that several symbionts are present, each using different fixation pathways.

## Conclusions

Our study encompassed several dominant species collected from each of three sites. Although local conditions were different at each site, nutritional relationships and trophic structures were similar for the same species across locations. However, for the two species collected in more than one site (*R*. *vandoverae* and *M*. *lauensis*), there were significant differences in the isotopic signatures between sites. First, chemosynthetic bacteria were the major primary source of carbon in this ecosystem. Second, the dominant species, including shrimps, galatheids, mussels and gastropods, at these hydrothermal vents were not apparently selective in their food choices, which may be due to the limited range of food sources. As primary consumers, mussels mainly obtained energy from symbiotic bacteria that used RuBisCO form I for the CBB cycle. Mussels also obtained some energy by filter-feeding, but in considerably lower amounts. Symbiotic polychaetes consumed mussels and symbiotic bacteria as prey. Like mussels, gastropods relied mainly on symbiotic bacteria for energy with some supplementary consumption of detritus. Tube worms obtained energy from symbiotic bacteria, but their bacterial species were different from those of the mussels. Finally, crustaceans were found to be omnivorous on chemosynthetic bacteria, detritus, or even animals.

Since most of the trophic structure relied on chemosynthetic bacteria and the bacteria relied on reduced compounds from the vent emissions, any disruption of vent emission by mining activities could have major consequences on the trophic structure and finally on vent ecosystem functioning. Moreover, our study suggested that there might be food relationship between animals, i.e., mussels and commensal scale worms. In addition, other members of the vent community must be considered in order to obtain a more comprehensive picture of the vent trophic network. Therefore, to evaluate the potential impacts of deep sea mining, it is necessary to study not only the direct destruction of the organisms attached to the mineral rocks but also the cascade effects on the food web of the whole ecosystem. Furthermore, the dense communities attached to rocks formed several microenvironments and presented high biodiversity. Although the relationship of interdependence among organisms in the microenvironment is not clear, there was no doubt that mining had effects on these microenvironments. Before exploiting deep-sea resources, further systematic investigations concerning the protection of deep-sea ecosystems are deeded.

## Supporting information

S1 Table Stable isotope results. (XLSX)

## References

[1] Le Bris N, Arnaud-Haond S, Beaulieu S, Cordes E, Hilario A, Rogers A, et al Hydrothermal Vents and Cold Seeps UN (Ed.). First Global Integrated Marine Assessment, 2016, 18.

[2] Reid WD, Sweeting CJ, Wigham BD, Zwirglmaier K, Hawkes JA, McGill RA, et al Spatial differences in East Scotia ridge hydrothermal vent food webs: influences of chemistry, microbiology and predation on trophodynamics. PLoS ONE. 2013;8(6):e65553 10.1371/journal.pone.0065553 23762393 10.1371/journal.pone.0065553 PMC3676328

[3] Demina L, Galkin S. On the role of abiogenic factors in the bioaccumulation of heavy metals by the hydrothermal fauna of the Mid-Atlantic Ridge. Oceanology. 2008;48(6):784–797.

[4] Fabri MC, Bargain A, Briand P, Gebruk A, Fouquet Y, Morineaux M, et al The hydrothermal vent community of a new deep-sea field, Ashadze-1, 12°58'N on the Mid-Atlantic Ridge. J Mar Biol Assoc U.K.. 2011; (91): 1–13.

[5] Gena K. Deep sea mining of submarine hydrothermal deposits and its possible environmental impact in Manus Basin, Papua New Guinea. Procedia Earth Planet. Sci. 2013;6:226–233. 10.1016/j.proeps.2013.01.031

[6] Beaulieu SE, Baker ET, German CR, Maffei A. An authoritative global database for active submarine hydrothermal vent fields. Geochem Geophys Geosyst. 2013;14(11):4892–4905. 10.1002/2013gc004998

[7] Collins PC, Kennedy B, Copley J, Boschen R, Fleming N, Forde J, et al VentBase: Developing a consensus among stakeholders in the deep-sea regarding environmental impact assessment for deep-sea mining–A workshop report. Mar Policy. 2013;42:334–336. 10.1016/j.marpol.2013.03.002

[8] Lonsdale P. Clustering of suspension-feeding macrobenthos near abyssal hydrothermal vents at oceanic spreading centers. Deep Sea Res Part 2 Top Stud Oceanogr. 1977;24(9):857.

[9] Rau GH, Hedges JI. Carbon-13 depletion in a hydrothermal vent mussel: suggestion of a chemosynthetic food source. Science. 1979;203(4381):648–649. 10.1126/science.203.4381.648 17813375 10.1126/science.203.4381.648

[10] Rau GH. Low ^15^N/^14^N in hydrothermal vent animals: ecological implications. Nature. 1981;289(5797):484–485.

[11] Knittel K, Boetius A. Anaerobic oxidation of methane: progress with an unknown process. Annu Rev Microbiol. 2009;63:311–334. 10.1146/annurev.micro.61.080706.093130 . 19575572 10.1146/annurev.micro.61.080706.093130

[12] Inagaki F, Kuypers MM, Tsunogai U, Ishibashi J, Nakamura K, Treude T, et al Microbial community in a sediment-hosted CO_2_ lake of the southern Okinawa Trough hydrothermal system. Proc Natl Acad Sci USA. 2006;103(38):14164–14169. 10.1073/pnas.0606083103 . 16959888 10.1073/pnas.0606083103 PMC1599929

[13] Fisher C. Chemoautotrophic and methanotrophic symbioses in marine invertebrates. Rev. Aquat. Sci. 1990;2(3–4):399–436.

[14] Jannasch HW. Chemosynthetically sustained ecosystems in the deep sea In: Schlegel H.G., Bowien B. (Eds.), Autotrophic Bacteria. Springer Verlag, Berlin 1989: 147–166.

[15] Orcutt BN, Sylvan JB, Knab NJ, Edwards KJ. Microbial ecology of the dark ocean above, at, and below the seafloor. Microbiol Mol Biol Rev. 2011;75(2):361–422. 10.1128/MMBR.00039-10 . 21646433 10.1128/MMBR.00039-10 PMC3122624

[16] Dubilier N, Bergin C, Lott C. Symbiotic diversity in marine animals: the art of harnessing chemosynthesis. Nat Rev Microbiol. 2008;6(10):725–740. 10.1038/nrmicro1992 18794911 10.1038/nrmicro1992

[17] Childress JJ, Fisher C, Brooks J, Kennicutt M, Bidigare R, Anderson A. A methanotrophic marine molluscan (Bivalvia, Mytilidae) symbiosis: mussels fueled by gas. Science. 1986;233(4770):1306–1308. 10.1126/science.233.4770.1306 17843358 10.1126/science.233.4770.1306

[18] Dover CLV, Fry B. Stable isotopic compositions of hydrothermal vent organisms. Mar Biol. 1989;102(2):257–263.

[19] Fisher CR, Childress JJ, Macko SA, Brooks JM. Nutritional interactions in Galapagos Rift hydrothermal vent communities: inferences from stable carbon and nitrogen isotope analyses. Mar Ecol Prog Ser. 1994;103(1–2):45–55.

[20] Sweetman AK, Levin LA, Rapp HT, Schander C. Faunal trophic structure at hydrothermal vents on the southern Mohn’s Ridge, Arctic Ocean. Mar Ecol Prog Ser. 2013;473:115–131. 10.3354/meps10050

[21] Comeault A, Stevens CJ, Juniper SK. Mixed photosynthetic-chemosynthetic diets in vent obligate macroinvertebrates at shallow hydrothermal vents on Volcano 1, South Tonga Arc-evidence from stable isotope and fatty acid analyses. Cah. Biol. 3 2010;51(4):351–359.

[22] Wang TW, Chan TY, Chan BKK. Trophic relationships of hydrothermal vent and non-vent communities in the upper sublittoral and upper bathyal zones off Kueishan Island, Taiwan: a combined morphological, gut content analysis and stable isotope approach. Mar Biol. 2014;161(11):2447–2463. 10.1007/s00227-014-2479-6

[23] Tarasov VG, Gebruk AV, Mironov AN, Moskalev LI. Deep-sea and shallow-water hydrothermal vent communities: Two different phenomena? Chem Geol. 2005;224(1–3):35–39. 10.1016/j.chemgeo.2005.07.021

[24] Bergquist DC, Eckner JT, Urcuyo IA, Cordes EE, Hourdez S, Macko SA, et al Using stable isotopes and quantitative community characteristics to determine a local hydrothermal vent food web. Mar Ecol Prog Ser. 2007;330:49–65.

[25] Colaço A, Dehairs F, Desbruyères D. Nutritional relations of deep-sea hydrothermal fields at the Mid-Atlantic Ridge: a stable isotope approach. Deep Sea Res Part 1 Oceanogr Res Pap. 2002;49(2):395–412.

[26] Michener RH, Les K. Stable Isotope Ratios as Tracers in Marine Food Webs: An Update Stable Isotopes in Ecology and Environmental Science, Second Edition Blackwell Publishing Ltd, 2007:238–282.

[27] Minagawa M, Wada E. Stepwise enrichment of ^15^N along food chains: Further evidence and the relation between δ^15^N and animal age. Geochim Cosmochim Acta. 1984;48(5):1135–1140.

[28] Peterson BJ, Fry B. Stable isotopes in ecosystem studies. Annu Rev Ecol Syst. 1987;18(1):293–320.

[29] Kroopnick PM. The distribution of ^13^C of ΣCO_2_ in the world oceans. Deep Sea Res A. 1985;32(1):57–84.

[30] Kroopnick P, Weiss R, Craig H. Total CO_2_, ^13^C, and dissolved oxygen-^18^O at Geosecs II in the North Atlantic. Earth Planet Sci Lett. 1972;16(1):103–110.

[31] Duplessy JC, Shackleton NJ, Fairbanks RG, Labeyrie L, Oppo D, Kallel N. Deepwater source variations during the last climatic cycle and their impact on the global deepwater circulation. Paleoceanography. 1988;3(3):343–360.

[32] Feng D, Cheng M, Kiel S, Qiu JW, Yang Q, Zhou H, et al Using *Bathymodiolus* tissue stable carbon, nitrogen and sulfur isotopes to infer biogeochemical process at a cold seep in the South China Sea. Deep Sea Res Part 1 Oceanogr Res Pap. 2015;104:52–59. 10.1016/j.dsr.2015.06.011

[33] Brooks JM, Kennicutt M, Fisher C, Macko S, Cole K, Childress J, et al Deep-sea hydrocarbon seep communities: evidence for energy and nutritional carbon sources. Science. 1987;238(4830):1138–1142. 10.1126/science.238.4830.1138 17839368 10.1126/science.238.4830.1138

[34] Whiticar MJ, Faber E, Schoell M. Biogenic methane formation in marine and freshwater environments: CO_2_ reduction vs. acetate fermentation—isotope evidence. Geochim Cosmochim Acta. 1986;50(5):693–709.

[35] Barry JP, Buck KR, Kochevar RK, Nelson DC, Fujiwara Y, Goffredi SK, et al Methane-based symbiosis in a mussel, *Bathymodiolus platifrons*, from cold seeps in Sagami Bay, Japan. Invertebr Biol. 2002;121(1):47–54.

[36] Levin LA, James DW, Martin CM, Rathburn AE, Harris LH, Michener RH. Do methane seeps support distinct macrofaunal assemblages? Observations on community structure and nutrition from the northern California slope and shelf. Mar Ecol Prog Ser. 2000;208(1):21–39.

[37] Herzig PM, Hannington MD, Arribas A Jr. Sulfur isotopic composition of hydrothermal precipitates from the Lau Back-Arc: implications for magmatic contributions to seafloor hydrothermal systems. Mineralium Deposita. 1998;33(3):226–237.

[38] Reid WDK, Wigham BD, McGill RAR, Polunin NVC. Elucidating trophic pathways in benthic deep-sea assemblages of the Mid-Atlantic Ridge north and south of the Charlie-Gibbs Fracture Zone. Mar Ecol Prog Ser. 2012;463:89–103. 10.3354/meps09863

[39] Portail M, Olu K, Dubois SF, Escobar-Briones E, Gelinas Y, Menot L, et al Food-web complexity in Guaymas Basin hydrothermal vents and cold seeps. PloS ONE. 2016;11(9):e0162263 10.1371/journal.pone.0162263 . 27683216 10.1371/journal.pone.0162263 PMC5040445

[40] Bojar AV, Lécuyer C, Bojar HP, Fourel F, Vasile Ş. Ecophysiology of the hydrothermal vent snail *Ifremeria nautilei* and *barnacle Eochionelasmus ohtai manusensis*, Manus Basin, Papua New Guinea: insights from shell mineralogy and stable isotope geochemistry. Deep Sea Res Part 1 Oceanogr Res Pap. 2018;133:49–58.

[41] Thal J, Tivey M, Yoerger D, Jöns N, Bach W. Geologic setting of PACManus hydrothermal area—High resolution mapping and in situ observations. Mar Geol. 2014;355:98–114. 10.1016/j.margeo.2014.05.011

[42] Hashimoto J, Furuta M. A new Species of *Bathymodiolus* (Bivalvia: Mytilidae) from hydrothermal vent communities in the Manus Basin, Papua New Guinea. Venus. 2007;66(1–2): 57–68.

[43] Galkin SV. Megafauna associated with hydrothermal vents in the Manus Back-Arc Basin (Bismarck Sea). Mar Geol. 1997;142(97):197–206.

[44] Both R, Crook K, Taylor B, Brogan S, Chappell B, Frankel E, et al Hydrothermal chimneys and associated fauna in the Manus Back–Arc basin, Papua New Guinea. EOS Trans AGU. Eos (Washington DC). 2013;67(21):489–90.

[45] Hashimoto J. Hydrothermal vent communities in the Manus Basin, Papua New Guinea: results of the BIOACCESS cruises '96 and '98. Inter Ridge News. 1999;8(2):12–18.

[46] Zhang X, Du Z, Zheng R, Luan Z, Qi F, Cheng K, et al Development of a new deep-sea hybrid Raman insertion probe and its application to the geochemistry of hydrothermal vent and cold seep fluids. Deep Sea Res Part 1 Oceanogr Res Pap. 2017; 123:1–12.

[47] Duperron S. The diversity of deep-sea mussels and their bacterial symbioses. The vent and seep biota. Springer, Dordrecht, 2010; 33:137–167. 10.1007/978-90-481-9572-5_6

[48] Duperron S, Guezi H, Gaudron SM, Pop Ristova P, Wenzhofer F, Boetius A. Relative abundances of methane- and sulphur-oxidising symbionts in the gills of a cold seep mussel and link to their potential energy sources. Geobiology. 2011;9(6):481–491. 10.1111/j.1472-4669.2011.00300.x . 21978364 10.1111/j.1472-4669.2011.00300.x

[49] Nelson DC, Hagen KD. Physiology and biochemistry of symbiotic and free-living chemoautotrophic sulfur bacteria. Am Zool. 1995;35(2):91–101.

[50] Cavanaugh CM. Symbioses of chemoautotrophic bacteria and marine invertebrates from hydrothermal vents and reducing sediments. Bull. Biol. Soc. Wash. 1985:373–388.

[51] Mccollom TM, Shock EL. Geochemical constraints on chemolithoautotrophic metabolism by microorganisms in seafloor hydrothermal systems. Geochim Cosmochim Acta. 1997;61(61):4375–4391. 11541662 10.1016/s0016-7037(97)00241-x

[52] Hugler M, Sievert SM. Beyond the Calvin cycle: autotrophic carbon fixation in the ocean. Ann Rev Mar Sci. 2011;3:261–289. 10.1146/annurev-marine-120709-142712 . 21329206 10.1146/annurev-marine-120709-142712

[53] Robinson JJ, Scott KM, Swanson ST, O'Leary MH, Horken K, Tabita FR, et al Kinetic isotope effect and characterization of form II RubisCO from the chemoautotrophic endosymbionts of the hydrothermal vent tubeworm *Riftia pachyptila*. Limnol Oceanogr. 2003;48(48):48–54.

[54] Campbell BJ, Cary SC. Abundance of reverse tricarboxylic acid cycle genes in free-living microorganisms at deep-sea hydrothermal vents. Appl Environ Microbiol. 2004;70(10):6282–6289. 10.1128/AEM.70.10.6282-6289.2004 . 15466576 10.1128/AEM.70.10.6282-6289.2004 PMC522104

[55] Suzuki Y, Sasaki T, Suzuki M, Nogi Y, Miwa T, Takai K, et al Novel chemoautotrophic endosymbiosis between a member of the Epsilonproteobacteria and the hydrothermal-vent gastropod *Alviniconcha aff*. *hessleri* (Gastropoda: Provannidae) from the Indian Ocean. Appl Environ Microbiol. 2005;71(9):5440–5450. 10.1128/AEM.71.9.5440-5450.2005 . 16151136 10.1128/AEM.71.9.5440-5450.2005 PMC1214688

[56] House CH, Schopf JW, Stetter KO. Carbon isotopic fractionation by Archaeans and other thermophilic prokaryotes. Org Geochem. 2003;34(3):345–356. 10.1016/s0146-6380(02)00237-1

[57] Wirsen CO, Sievert SM, Cavanaugh CM, Molyneaux SJ, Ahmad A, Taylor LT, et al Characterization of an autotrophic sulfide-oxidizing marine *Arcobacter* sp. that produces filamentous sulfur. Appl Environ Microbiol. 2002;68(1):316–325. 10.1128/AEM.68.1.316-325.2002 11772641 10.1128/AEM.68.1.316-325.2002 PMC126556

[58] Cuy RD, Fogel ML, Berry JA. Photosynthetic fractionation of the stable isotopes of oxygen and carbon. Plant Physiol, 1993: 101(1): 37–47. 12231663 10.1104/pp.101.1.37 PMC158645

[59] Roeske CA, O'Leary MH. Carbon isotope effects on enzyme-catalyzed carboxylation of ribulose bisphosphate. Biochem. 2002;23(25):6275–6284. 10.1021/bi00328a005 3924094

[60] Desbruyères D, Alayse-Daneta AM. Deep-sea hydrothermal communities in Southwestern Pacific back-arc basins (the North Fiji and Lau Basins): Composition, microdistribution and food web. Mar Geol. 1994;116(1–2):227–242.

[61] Comitet T, Krylova EM. *Bathymodiolus* (Bivalvia: Mytilidae) from hydrothermal vents on the Azores triple junction and the Logatchev hydrothermal field, Mid-Atlantic Ridge. Veliger. 1999;42(3):218–248.

[62] Pennec ML, Beninger PG, Herry A. Feeding and digestive adaptations of bivalve molluscs to sulphide-rich habitats. Comp Biochem Physiol A Physiol. 1995;111(2):183–189.

[63] Page HM, Fisher CR, Childress JJ. Role of filter-feeding in the nutritional biology of a deep-sea mussel with methanotrophic symbionts. Mar Biol. 1990;104(2):251–257.

[64] Desbruyères D, Gaill F, Laubier L, Fouquet Y. Polychaetous annelids from hydrothermal vent ecosystems: An ecological overview. Bull. Biol. Soc. Wash. 1985;67(11):103–116.

[65] Waka SO, Okoshi K, Fujikura K, Fujiwara Y. Polychaetes inhabiting the mantle cavity of deep-sea bivalves in the ryukyu islands region and the japan trench (preliminary report). Nihon Bentosu Gakkai Shi. 2003;58:70–76.

[66] Dover CLV, Fry B, Grassle JF, Humphris S, Rona PA. Feeding biology of the shrimp *Rimicaris exoculata* at hydrothermal vents on the Mid-Atlantic Ridge. Mar Biol. 1988;98(2):209–216.

[67] Pond DW, Gebruk A, Southward EC, Southward AJ, Fallick AE, Bell MV, et al Unusual fatty acid composition of storage lipids in the bresilioid shrimp *Rimicaris exoculata* couples the photic zone with MAR hydrothermal vent sites. Mar Ecol Prog Ser. 2000;198(3):171–179.

[68] Gebruk AV, Southward EC, Kennedy H, Southward AJ. Food sources, behaviour, and distribution of hydrothermal vent shrimps at the Mid-Atlantic Ridge. J Mar Biol Assoc U.K. 2000;80(3):485–499.

[69] Streit K, Bennett SA, Van Dover CL, Coleman M. Sources of organic carbon for *Rimicaris hybisae*: Tracing individual fatty acids at two hydrothermal vent fields in the Mid-Cayman Rise. Deep Sea Res Part 1 Oceanogr Res Pap. 2015;100:13–20. 10.1016/j.dsr.2015.02.003

[70] Ponsard J, Cambon–Bonavita MA, Zbinden M, Lepoint G, Joassin A, Corbari L, et al Inorganic carbon fixation by chemosynthetic ectosymbionts and nutritional transfers to the hydrothermal vent host-shrimp *Rimicaris exoculata*. ISME J. 2013;7(1):96–109. 10.1038/ismej.2012.87 22914596 10.1038/ismej.2012.87 PMC3526180

[71] Escobar-Briones E, Morales P, Cienfuegos E, González M, Ciencias U. Carbon sources and trophic position of two abyssal species of Anomura, *Munidopsis alvisca* (Galatheidae) and *Neolithodes diomedeae* (Lithodidae). Contributions to the Study of East Pacific Crustaceans. 2002: 37–43.

[72] Phleger CF, Nelson MM, Groce AK, Cary SC, Coyne KJ, Nichols PD. Lipid composition of deep-sea hydrothermal vent tubeworm *Riftia pachyptila*, crabs *Munidopsis subsquamosa* and *Bythograea thermydron*, mussels *Bathymodiolus* sp. and limpets *Lepetodrilus* spp. Comp Biochem Physiol B Biochem Mol Biol. 2005;141(2):196–210. 10.1016/j.cbpc.2005.03.001 . 15893489 10.1016/j.cbpc.2005.03.001

[73] Pond DW, Dixon DR, Bell MV, Fallick AE, Sargent JR. Occurrence of 16:2(n-4) and 18:2(n-4) fatty acids in the lipids of the hydrothermal vent shrimps *Rimicaris exoculata* and *Alvinocaris markensis*: Nutritional and trophic implications. Mar Ecol Prog Ser. 1997;156(8):167–174.

[74] Rieley G, Dover CLV, Hedrick DB, Eglinton G. Trophic ecology of *Rimicaris exoculata*: a combined lipid abundance/stable isotope approach. Mar Biol. 1999;133(3):495–499.

[75] Hügler M, Petersen JM, Dubilier N, Imhoff JF, Sievert SM. Pathways of carbon and energy metabolism of the epibiotic community associated with the deep-sea hydrothermal vent shrimp *Rimicaris exoculata*. PloS ONE. 2011;6(1):e16018 10.1371/journal.pone.0016018 21249205 10.1371/journal.pone.0016018 PMC3017555

[76] Saito H, Hashimoto J. Characteristics of the fatty acid composition of a deep-sea vent gastropod, *Ifremeria nautilei*. Lipids. 2010;45(6):537–548. 10.1007/s11745-010-3436-x . 20549377 10.1007/s11745-010-3436-x

[77] Windoffer R, Giere O. Symbiosis of the hydrothermal vent gastropod *Ifremeria nautilei* (Provannidae) with endobacteria-structural analyses and ecological considerations. Biol Bull. 1997;193(3):381–392. 10.2307/1542940 28574764 10.2307/1542940

[78] Seston SL, Beinart RA, Sarode N, Shockey AC, Ranjan P, Ganesh S, et al Metatranscriptional response of chemoautotrophic *Ifremeria nautilei* endosymbionts to differing sulfur regimes. Front Microbiol. 2016;7:1074 Epub 2016/08/04. 10.3389/fmicb.2016.01074 . 27486438 10.3389/fmicb.2016.01074 PMC4949241

[79] Amano K, Jenkins RG. A new species of *Provanna* (Gastropoda: Provannidae) from an oligocene seep deposit in eastern hokkaido, Japan. Paleontol Res. 2013;17(4):325–329. 10.2517/1342-8144-17.4.325

[80] Sasaki T, Warén A, Kano Y, Okutani T, Fujikura K. Gastropods from recent hot vents and cold seeps: systematics, diversity and life strategies The vent and seep biota. Springer, Dordrecht, 2010: 169–254.

[81] Kiel S, Tyler P A, Vrijenhoek R C. The Vent and Seep Biota: Springer Netherlands 2010, 33.

